# Botulinum toxin type a within function-oriented management of post-stroke spasticity: a narrative review

**DOI:** 10.3389/fneur.2026.1921220

**Published:** 2026-10-12

**Authors:** Baoling Su, Yating Huang, Xiaotong Li, Kun Wang, Bing Bao

**Affiliations:** School of Health Science and Engineering, University of Shanghai for Science and Technology, Shanghai, China

**Keywords:** participation in daily life, botulinum toxin type a, clinical heterogeneity, functional goal stratification, individualized management, pain, positioning, post-stroke care needs

## Abstract

Post-stroke spasticity (PSS) is a heterogeneous complication of stroke that affects motor recovery, care needs, pain, positioning, and participation in daily life. Current management frameworks commonly stratify patients by time since stroke, but disease duration alone does not capture differences in neural mechanisms, residual voluntary control, functional priorities, or response to intervention. This narrative review searched PubMed, the Cochrane Library, and Wanfang databases from inception to May 2026, prioritizing randomized controlled trials, prospective cohort studies, systematic reviews, real-world studies, Goal Attainment Scaling research, the Upper Limb International Spasticity cohort series, best-practice guidance, and clinical trials including BEST and InTENSE. We synthesize evidence that PSS heterogeneity can be understood across central and peripheral mechanisms, temporal trajectories, and active versus passive functional consequences. Available studies suggest that active and passive functional goals differ in baseline functional profiles, outcome assessment, treatment response, and intervention requirements. However, patients sharing a functional goal type may still differ in residual voluntary control, neural and non-neural contributors to movement restriction, pain, mobility, and care dependency. Botulinum toxin type A is well aligned with focal tone reduction and passive goals, including pain relief, hygiene, and limb positioning. It may also support contracture-prevention strategies when modifiable neural muscle overactivity remains, whereas active functional gains usually require preserved motor potential and integration with task-oriented training, rehabilitation, and adjunctive modalities. We propose that functional goal type should complement time-based staging as a decision dimension rather than serve as a complete clinical phenotype. This combined framework may better align patient selection, intervention matching, outcome assessment, and longitudinal treatment planning. Prospective goal-stratified studies and standardized assessment tools are needed to test its clinical utility.

## 1 Introduction

Stroke is a leading cause of death and long-term disability worldwide. Global Burden of Disease 2021 projections indicate that stroke-related disability-adjusted life years will continue to increase up to 2050, with much of this burden occurring in low- and middle-income countries (1, 2). Post-stroke spasticity (PSS) is an important complication during motor recovery. Its pooled prevalence is approximately 25%, and the point prevalence among patients with paralysis or hemiparesis is approximately 39.5%. Disabling or severe spasticity occurs in about 9-10% of stroke survivors (3). PSS also increases caregiver burden and health-care costs, with direct medical costs reported to be about four times higher in stroke survivors with spasticity than in those without spasticity (4, 5).

Management strategies for PSS include focal injections of botulinum toxin type A (BoNT-A), oral antispastic medications, intrathecal baclofen, physical therapy, orthotic management, and neuromodulation (6–10). In practice and research, PSS is often staged by time since stroke, using acute, subacute, and chronic phases. This time-based framework is useful for standardizing clinical pathways, but it assumes that patients at similar post-stroke intervals have broadly similar impairment patterns and treatment priorities. Clinical practice shows otherwise. Patients within the same time window may differ substantially in lesion characteristics, baseline reserve, motor recovery potential, pain, care needs, and personal priorities (7–10).

Functional goal-oriented stratification offers a complementary way to address this heterogeneity. It distinguishes active goals, such as grasping, walking, transfers, and self-care, from passive goals, such as pain relief, hygiene, limb positioning, nursing care, and contracture prevention. Evidence from Goal Attainment Scaling (GAS) and the Upper Limb International Spasticity cohort series supports the clinical relevance of functional goal classification and its association with outcomes (10, 11). Accordingly, this review examines sources of PSS heterogeneity, analyzes limitations of time-based staging for individualized decision-making, synthesizes the rationale and evidence for functional goal-oriented stratification, and explores how different interventions may align with active and passive functional goals.

## 2 Methods

This narrative review was conducted by systematically searching for relevant literature in PubMed, Coch- rane Library, and Wanfang databases. We searched PubMed, the Cochrane Library, and Wanfang databases from inception to the end of May 2026. We conducted this narrative review using a search strategy that combined Medical Subject Headings (MeSH) and free-text words as keywords: [post-stroke spasticity] AND [goal attainment scaling] AND [botulinum toxin] AND for functional outcome which is spasticity management. The literature was screened based on the PRISMA guidelines as depicted in Figure 1. Randomized controlled trials, prospective cohort studies, systematic reviews, and real-world studies were prioritized. Evidence from GAS-based studies, the ULIS cohort series, and clinical trials such as BEST and InTENSE was also synthesized. This review first systematically addressed sources of PSS clinical heterogeneity based on the following literature search strategy at three levels: neurological mechanisms, temporal course, and patterns of functional impact (see details in the methods section).

**Figure 1:**
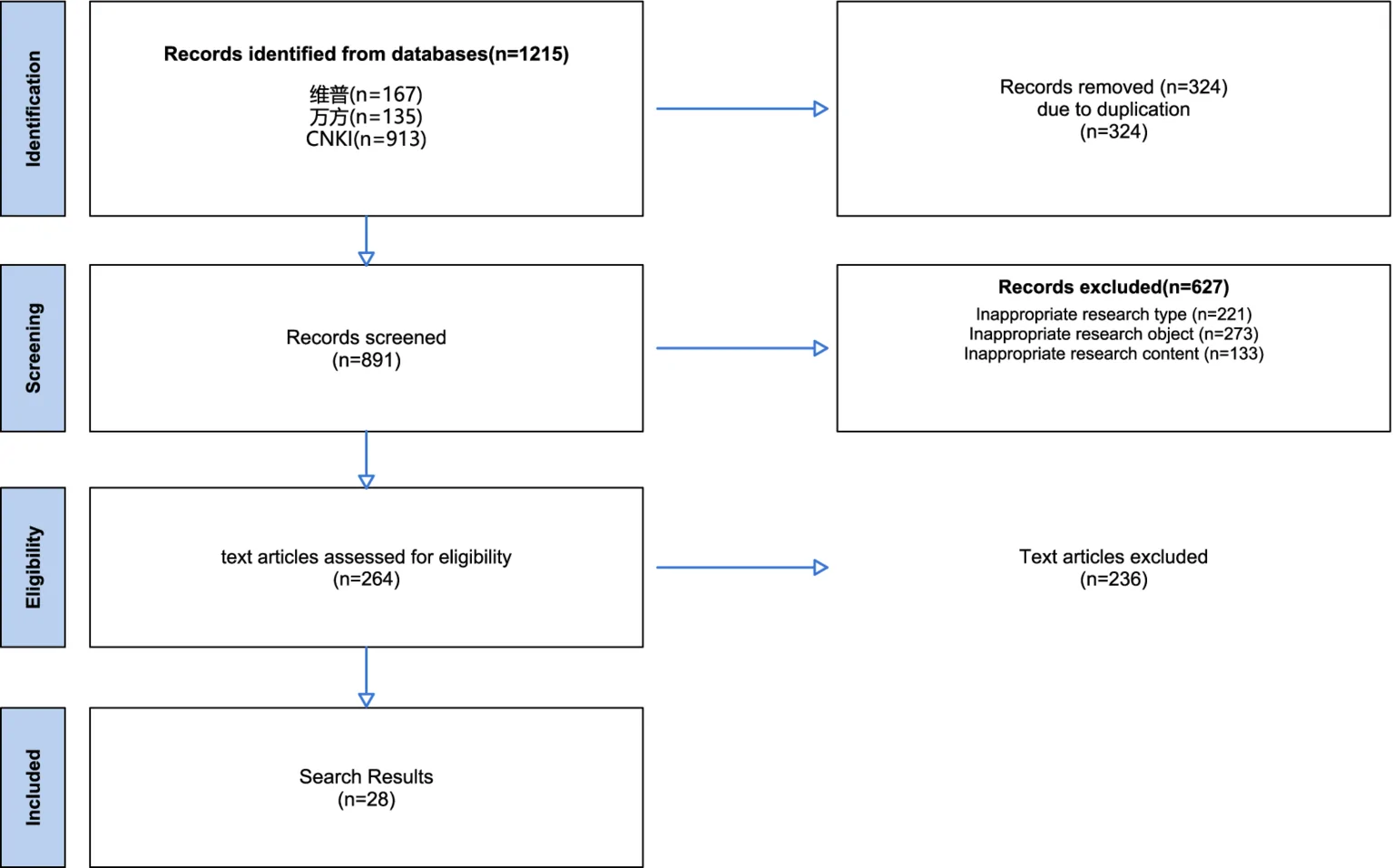
Literature screening flowchart.

## 3 Clinical heterogeneity of post-stroke spasticity

PSS is not a homogeneous clinical entity; it exhibits significant individual differences in neurological mechanisms, temporal course, severity distribution, and patterns of functional impact. This heterogeneity makes it difficult for a single standardized treatment protocol to meet the actual needs of all patients and poses challenges to the current staging framework centered on time intervals. An in-depth understanding of the sources and clinical significance of PSS heterogeneity is a prerequisite for constructing an individualized stratification management framework. The following sections analyze this heterogeneity from three perspectives: central and peripheral mechanisms, temporal course and natural history, and patterns of functional impact.

### 3.1 Central and peripheral mechanisms underlying PSS heterogeneity

PSS reflects a multilevel, multipathway pathophysiological cascade following central nervous system injury. Maladaptive plasticity mechanisms may contribute to the development of post-stroke spasticity (PSS) at the central level. Specifically, stroke-induced corticospinal tract (CST) injury reduces descending cortical inhibition of brainstem reticular structures, thereby reducing cortical inhibition of reticulospinal tract activity and increasing excitatory drive to spinal motor circuits. This provides an inappropriate net enhancement of excitatory input to spinal motoneurons and interneurons, which in turn contributes to hyperexcitability of even uninjured muscle stretch reflex arcs (12, 13). Importantly, evidence from EMG- EMG coherence suggests that motor overflow and spasticity could coexist in the same patient during the spasticity period but disappear in complete recoverers (14). This finding suggests that reticulospinal tract- related maladaptive reorganization may be more closely associated with the formation of spasticity than with simple processes indirectly compensating for loss of motor function (14). In addition, spinal segmental mechanisms such as reduced synaptic efficacy of Ia afferent fibers, weakened circuits mediated by Ib inhibitory interneurons, and decreased reciprocal inhibition may further facilitate the stretch reflex (13). The specific combinations of these central mechanisms differ among individuals depending on factors such as stroke location, lesion extent, and the nature of corticospinal tract involvement. Peripheral mechanisms are also major contributors to PSS heterogeneity. These muscle-level changes are encompassed by the concept of spastic myopathy and should not be regarded solely as late consequences of long-standing spasticity. Following stroke, paresis, reduced mobilization, and sustained positioning of muscles at short length may drive plastic changes that include diminished muscle-fiber cross-sectional area, a shift from oxidative toward glycolytic fibers, intramuscular connective-tissue accumulation, and fat infiltration (15, 16). Such changes may begin before a fixed contracture is established and interact with neural muscle overactivity and altered afferent input, promoting a cycle of pathological reflex hyperactivity, disuse muscle atrophy, and progressive structural shortening (16). Because this process progresses at different rates from person to person, the relative contributions of neural and structural components to increased muscle tone differ among patients. Clinically, limitation can therefore be considered along a continuum from neural muscle overactivity producing a dynamic deformity, through mixed neural and soft-tissue restriction, to fixed structural contracture. The term “dynamic contracture” is sometimes used for a posture-, task-, or velocity-dependent deformity with a substantial reversible neural component; here, “dynamic deformity” is used to distinguish this presentation from established tissue shortening. This distinction is clinically important because BoNT-A is expected to be most effective when a dynamic or mixed limitation retains a modifiable neural component, whereas it cannot by itself reverse passive range-of-motion loss primarily caused by fixed shortening of muscle and connective tissue (17). Even when structural contracture is established, residual neural muscle overactivity may remain a separate, goal-dependent treatment target. Consequently, assessment should distinguish residual dynamic overactivity, soft-tissue extensibility, and fixed structural limitation in order to define intervention targets, treatment expectations, and functional objectives. Nevertheless, the Modified Ashworth Scale (MAS), a frequently used clinical spasticity measurement tool based on resistance to passive movement, does not differentiate neural spasticity from structural contractures or demonstrate the actual influence of spasticity on functional activities (18). Muscle tone scores for individual muscles considered in isolation do not adequately characterize the relative neural and structural contributions to movement restriction (19). Beyond these mechanistic differences, individual variability in the temporal course and natural history of PSS also complicates time-based staging frameworks.

### 3.2 Individual differences in PSS onset timing and natural history

Although time-based staging frameworks are useful for standardizing clinical pathways and research design, they have clear limitations in explaining individual variation in treatment response. Onset timing and the evolution of PSS differ significantly among individuals, with onset reported from days to months after stroke (20). These limitations can be summarized in three main aspects: the relationship between disease duration and treatment response is nonlinear; post-stroke functional recovery trajectories are heterogeneous; and the evolution of spasticity does not necessarily parallel motor recovery. First, treatment response cannot be reliably predicted from disease duration alone. The early-BIRD real-world study included 303 patients with upper limb spasticity who were classified into early, intermediate, and late initiation groups according to the time from stroke to first BoNT-A treatment. The authors hypothesized that early initiators would show greater improvements in MAS scores for the elbow and wrist flexors. However, across repeated injection cycles, MAS scores for upper-limb flexor muscles decreased without significant differences among groups; the adjusted mean difference at the final visit between the early- and late-initiation groups was 0.15 and did not reach statistical significance (*p* = 0.55) (21). Although limited by the pitfalls of observational studies, including selection bias and residual confounding, this work suggests that predicting treatment response

solely from disease duration is overly simplistic. In addition, factors predicting spasticity severity at 3–6 months after stroke are numerous and are not limited to time since stroke; initial stroke severity, lesion location, and motor impairment also play significant roles in determining spasticity outcomes (22, 23). It is therefore implausible that time after injury alone can guide management of this potentially disabling complication. Second, functional recovery trajectories following stroke vary substantially. In a longitudinal analysis of disability trajectories during the first year after ischemic stroke, four patterns of functional evolution were identified: no significant disability, mild disability followed by recovery, severe disability improving to a moderate level, and persistent severe disability (24). This suggests that even at comparable disease stages, patients may exhibit different functional states and clinical rehabilitation courses that cannot be adequately captured by single time points. Third, spasticity formation and motor function recovery do not completely coincide. PSS is mainly associated with maladaptive plasticity in the context of upper motor neuron syndrome, whereas motor function recovery depends more on cortical reorganization and CST integrity (7, 25). The onset and time course of spasticity also follow a significant individual and non-uniform trajectory (26). Although the two processes interact, they follow distinct neurophysiological pathways. Thus, patients at the same disease stage may have different amounts of spasticity, impairment in voluntary motor control, structural contractures, and functional compensation. The lack of a time-based dimension for staging severely limits our ability to explain dissociations between spasticity severity and functional performance (27). Overall, although time-based staging frameworks aid in describing broad PSS evolution patterns, they have clear limitations for individual decision-making. These limitations indicate that additional stratification dimensions should be added to better capture patients’ functional status and treatment needs. Aside from temporal factors, the functional impact patterns of PSS also show pronounced heterogeneity that is closely related to treatment goals.

### 3.3 Heterogeneity of active and passive functional impairment

The functional impact of PSS also shows marked heterogeneity. Active function mainly indicates the ability of patients to use affected limbs for tasks such as grasping, feeding, and writing, whereas passive function more often reflects limb positioning, dressing, hygiene care, nursing, and pain control. The degree to which these two functional categories are impaired in an individual directly influences rehabilitation goals, intervention priorities, and outcome evaluation (10, 28).

Active functional impairment reflects deficits such as decreased voluntary motor capacity in the affected limbs, including poor upper-limb grasp and manipulation, fine-motor deficits in the hand, and lower-limb gait abnormalities such as knee hyperextension, equinovarus deformity, or circumduction. In the ULIS cohort series, both active and passive functional goals were commonly selected as primary goals when patients with PSS received BoNT-A treatment; the two goal types differed in baseline motor function, spasticity severity, and treatment expectations (10, 11, 28, 29). By contrast, passive functional impairment presents as caregiving challenges, abnormal limb positioning, contractures, and spasticity-related pain. Overall, these patients demonstrate more severe motor impairment and comorbidities, with less potential for active functional recovery. The passive care difficulty score was significantly positively correlated with the composite spasticity score in patients with upper-limb spasticity in a nursing-home study (r_s_ = 0.85, *p* < 0.001) (30). A subgroup analysis of ULIS-II showed that patients whose primary goal was passive function could still attain clinically relevant goals after BoNT-A treatment, although their baseline motor function was lower than that of patients with an active functional goal (28). Staging based on muscle tone severity or time since stroke provides only an indirect functional evaluation because it does not reflect patients’ actual rehabilitation needs, whereas goal classification provides a theoretical rationale for functional goal-oriented stratification (28, 31). Based on this evidence of heterogeneity, the following sections further explore the theoretical rationale and existing evidentiary foundation for functional goal-oriented stratification.

## 4 Theoretical rationale and research evidence for functional goal-oriented stratification

Significant heterogeneity in PSS neurological mechanisms, temporal course, and functional impact patterns makes staging frameworks relying solely on time intervals inadequate for fully reflecting patients’ actual functional status and treatment needs. At the same disease stage, different patients may face vastly different rehabilitation goals: some strive to restore independent walking and other active functions, while others prioritize improving limb positioning and reducing caregiver burden as primary demands. Functional goal-oriented stratification does not completely replace traditional time-based staging but rather introduces patients’ realistic functional status and treatment goal types as complementary dimensions to existing temporal frameworks, thereby enhancing specificity and individualization of clinical decision-making.

### 4.1 Current time-based staging framework and its theoretical basis

Current PSS management commonly relies on staging models based primarily on post-stroke time windows, thereby dividing the disease course into acute, subacute, and chronic phases. This time-based staging framework is convenient for depicting general patterns of post-stroke recovery trajectories. It aids the evaluation and determination of protocols for screening, rehabilitation, antispasticity treatment, and follow-up, as well as the standardization of therapeutic efficacy assessments in scientific research. The framework is largely based on the post-stroke neural repair hypothesis of a “window period,” which proposes that neuroplasticity caused by post-stroke injury is relatively active in the early post-stroke phase: approximately 60–80% of motor function recovery usually occurs in the first3–6 months and gradually reaches a relative plateau around 3 and/or 6 months after stroke onset (25, 32). This perspective has led current stroke rehabilitation guidelines to focus on early intervention (33, 34). Similarly, motor rehabilitation guidelines from the European Stroke Organisation (ESO) take a disorder-stage classification approach and propose assessing task capacity while integrating treatment recommendations for each phase into an overarching framework (33, 35). In like manner, the China Stroke Association guidelines for the clinical management of ischemic cerebrovascular diseases also use a time-based management framework (36). It is worth noting that although time-based staging primarily addresses the question of which post-stroke stage the patient currently occupies, it cannot sufficiently explain clinical presentations such as functional status at rest and during movement, spasticity phenotype, and treatment priorities. Patients in the same time window may have very different active motor potential, spasticity grades, pain levels, or functional mobility and care dependency (31), yet may share similar treatment goals. Recent international guidelines and expert consensus have shifted away from placing absolute time labels at the forefront of evaluation, suggesting that clinicians should also assess whether spasticity has resulted in active functional limitation, pain, impaired range of motion or posture, or care difficulty. Dynamic management concepts focused on symptoms and signs have similarly attracted recent interest (17, 19, 37, 38). Despite the strengths of time-based staging frameworks, their limitations in accounting for individual differences in treatment response have also been highlighted by an emerging body of clinical research.

### 4.2 Limitations of time-based staging in individualized decision-making

The relative treatment hyporesponsiveness of BoNT-A in patients with PSS cannot be predicted simply on the basis of disease duration, as suggested by real-world BoNT-A treatment studies. The early-BIRD study randomized 303 patients with upper-limb spasticity according to the time from stroke to first BoNT-A treatment into three groups: early (≤6 months), intermediate (7–12 months), and late initiation (≥12 months). The original hypothesis that MAS score improvements for elbow and wrist flexor muscles might be greater in early initiators was not fulfilled, as MAS scores decreased over repeated injection cycles in all three groups and the adjusted mean difference at the final visit between the early- and late-initiation groups was only 0.15 (not statistically significant, *p* = 0.55) (21). This observational real-world study is subject to selection bias and residual confounding and cannot infer causation, but its results discourage the overly simplistic assumption that “the longer the disease duration, the poorer the treatment response.” Evidence-based medicine requires comprehensive judgment, including functional status and treatment goals. Stroke is highly heterogeneous in its functional recovery trajectory. A longitudinal cohort analysis of disability trajectories during the first year after ischemic stroke identified four patterns of functional evolution: (i) no significant disability, (ii) mild disability to recovery, (iii) severe to moderate disability, and (iv) persistent severe disability (24). This indicates that functional outcomes after stroke do not follow a single uniform timeline and indirectly suggests the need to reconsider a single time-based staging of post-stroke recovery. Moreover, the development of spasticity and recovery of motor function are not entirely synchronous events. PSS formation is more closely associated with maladaptive plasticity in upper motor neuron syndrome, whereas motor recovery depends more on cortical reorganization and CST integrity (7, 25). Despite their interactions, the two processes do not change in parallel. The onset and time course of spasticity also follow a significant individual and non-uniform trajectory (26). Thus, patients at the same disease stage may display varying degrees of spasticity, impairment in voluntary motor control, structural contracture, and functional compensation. These dissociations represent the main limitations of staging spasticity severity solely on the basis of post-stroke time (27). In conclusion, time-based staging frameworks offer several standardization advantages; however, these models have limited ability to explain an individual’s functional status, treatment goals, or responses to intervention. This provides a rationale for introducing functional goals as an additional stratification dimension.

### 4.3 Theoretical rationale for incorporating the functional goal dimension

Functional goals can offset some shortcomings of time-based staging. Time-based staging can provide insight into patients’ relative recovery stage, but it does not adequately explain functional differences among patients within a similar time window. Chronic-phase patients may still improve walking, positioning, or ease of care through decreased focal tone in relatively spared muscles, whereas some subacute-phase patients may have severe motor control impairment, pain, or contracture risk, making active functional recovery an impractical priority at that time (37, 39). Functional goals emphasize the clinical consequences of spasticity rather than merely reporting muscle tone severity. For patients who preserve at least a limited degree of voluntary motor function, treatment objectives may primarily involve activities of daily living (ADL), such as grasping, feeding, assisted transfers, or ambulation. For patients with greater motor dysfunction or care dependency, treatment targets may be more closely related to pain management, limb positioning, optimizing joint range of motion (ROM), and preventing soft-tissue contracture. This division of active and passive functional goals is also reflected in clinical research, with the BEST trial using it as the main basis for efficacy assessment (40). This stratification method also elucidates differences in intervention allocation. When the main outcome is active functional improvement, reducing muscle tone usually provides only part of the conditions needed for motor execution, and task-oriented training is required to promote translation into function (41). If the goals are mainly pain relief, posture management, or ease of nursing care, focal antispastic treatment, stretching techniques, orthotics, or positioning management may have more immediate clinical relevance. Evaluating efficacy by changes in muscle tone scores or overall goal attainment rates without accounting for different goal types may mask variability in treatment benefits across patients (37). Therefore, the significance of functional goal-oriented stratification lies in integrating disease-course information provided by time-based staging with patients’ current functional problems. However, functional goal type is a complementary decision dimension rather than a complete patient phenotype and should be interpreted alongside residual voluntary motor capacity, neural and non-neural contributors to movement restriction, pain, mobility, and care dependency. Incorporating temporal factors and functional goal types can therefore support a more comprehensive clinical characterization of PSS heterogeneity and provide a logical starting point for evaluating evidence for different goal types. After establishing the theoretical framework, it is necessary to examine the empirical foundations for functional goal-oriented stratification in existing clinical research and identify remaining evidence gaps.

## 5 From goal classification to dynamic stratification: existing evidence and its limitations

Over the last decade, multicenter studies on GAS, functional goal classification systems, and goal-oriented treatment have provided important data for understanding the classifications, distributions, and clinical implications of treatment goals in post-stroke spasticity (PSS). Nonetheless, existing evidence has centered on cross-sectional goal descriptions, single-cycle goal attainment rates, or real-world cohort analyses, with few coherent dynamic stratification frameworks to inform multi-cycle treatment options.

### 5.1 Development and application of PSS functional goal classification systems

The management of PSS has gradually shifted from simple symptom quantification toward a functional needs-oriented approach. Early studies mostly used improvement in the Modified Ashworth Scale (MAS) score as the primary efficacy outcome (19, 42, 43). In developing goal-oriented treatment concepts, researchers became increasingly aware that treatment goals differ substantially in nature: some are aimed at patients’ ability to perform activities of daily living, while others describe caregivers’ convenience (formerly categorized as passive care) when providing routine assistance; still others focus mainly on alleviating pain, improving trunk posture, or preventing contractures. Combining these various goal types into single outcome labels may conceal variability in implementation ease, treatment benefit, and clinical relevance across goal types (31, 44, 45). In the prospective ULIS-III cohort study of population characteristics and treatment factors in 953 patients undergoing BoNT-A treatment for upper-limb spasticity with Goal Attainment Scaling (GAS), Turner-Stokes et al. demonstrated that passive functional goals were achieved at a significantly higher rate than active functional goals for both self-care and mobility (86.6% vs. 71.4%). This suggests that goal attainment is not a homogeneous endpoint; moreover, passive and active functional goals differ in their likelihood of attainment and should not be treated as equivalent outcome categories (29, 31). The International Classification of Functioning, Disability and Health (ICF) provides an essential basis for standardizing functional goal classification systems related to PSS, which in turn expands spasticity treatment goals from basic physiological indicators, such as muscle tone or range of motion, to comprehensive measures including daily activity, ease of care, and pain control. Ashford et al. performed a systematic analysis of treatment goals for patients with lower-limb spasticity, classifying 127 patients into six categories across two main domains (46) and laying the empirical foundation for classifying functional goals in spasticity. More recently, meta-analyses have provided additional evidence for the international generalizability of this classification system (31). However, the ICF framework itself primarily provides a language system for describing functional status and treatment goals at specific time points; it does not provide specific longitudinal decision rules regarding how goal types dynamically evolve throughout treatment. At the clinical application level, the Goal-Oriented Facilitated Approach to Spasticity Treatment (GO-FAST) further links functional goal types with common spasticity motor patterns and recommended injection target muscles, transforming goal classification from a descriptive tool into one with clinical decision-support value (47). As early as 2006, Turner-Stokes et al. demonstrated that GAS could effectively quantify functional benefits after BoNT-A treatment, particularly improvements that are difficult to capture with traditional muscle tone scales (48). This finding suggests that different functional goals may correspond to different spasticity patterns, anatomical targets, and injection strategies. However, GO-FAST is primarily applied to goal setting and injection planning within the current visit cycle, while clear guidance is limited regarding whether patients’ goal types transition across multiple treatment cycles and when treatment emphasis should be adjusted (37, 49). This suggests that existing goal classification tools, although clinically valuable, require further development to form longitudinal dynamic stratification frameworks.

### 5.2 Association between functional goals and baseline functional status

The distribution of functional goal types is not random but systematically associated with (i) the affected anatomical region and (ii) baseline functional status. To this end, Ashford et al. integrated data from the LeAM study, related datasets on ankle contractures, and cohorts involved with the ULIS-III meta-analysis (including 1,207 patients), showing that a significantly greater proportion of spasticity-related goals were passive functional goals among upper-limb patients (37%) than among lower-limb patients (22%), whereas nearly half of the goals involving lower-limb patients were active functional goals (44%), compared with 13% in the upper limb (31) (Table 1). Such a contrast is not hard to fathom: the upper limb serves tasks requiring fine manipulation in activities of daily living; when voluntary movement fails, caregiving goals such as hand hygiene, dressing, and positioning quickly eclipse all else. Moreover, the core function of the lower limb during weight-bearing and mobility leads to more frequent active task goals such as walking, transfers, and postural control (28, 29).

**Table 1:** Distribution of functional goal types in patients with upper- and lower-limb spasticity from the meta-analysis by Ashford et al.

Functional goal type	Upper-limb spasticity (% of goals)	Lower-limb spasticity (% of goals)	Clinical implication
Passive functional goals	37%	22%	Upper limb spasticity more commonly affects hand hygiene, dressing, positioning, and care procedures
Active functional goals	13%	44%	Lower-limb spasticity is more closely associated with active tasks such as walking, transfers, standing, and postural control
Pain management goals	22%	15%	Pain control is an important goal for both upper and lower limb spasticity
Contracture prevention goals	15%	17%	Contracture prevention shows stable demand in both upper and lower limb spasticity management
Involuntary movement goals	12%	Not listed as a major category	A notable proportion of upper-limb goals involves involuntary-movement control needs

Compiled from the results of Ashford et al. (31). The table displays overall trends in treatment-goal distribution for patients with different affected regions.

More detailed associations between goal types and patients’ baseline status were identified in a post-hoc subgroup analysis of ULIS-II. Fheodoroff et al. found that the 456 patients with upper-limb spasticity enrolled for single-cycle BoNT-A treatment were generally more severely impaired, had a longer disease duration, and had greater caregiving needs; however, baseline motor function was better among patients whose primary goal was active function (28). This implies that functional goals are, to some degree, an objective reflection of patients’ current motor function and rehabilitation needs rather than merely subjective desires. This differentiation is clinically significant. In the BEST trial, Ward et al. used the active functional goal attainment rate as the primary endpoint to compare the efficacy of BoNT-A with placebo for upper- limb spasticity; the results showed no statistically significant difference between groups. However, for the secondary endpoint of passive functional goal attainment, the BoNT-A group demonstrated a significant advantage (60.6% vs. 38.6%, OR = 2.46) (40). This “negative primary endpoint, positive secondary endpoint” pattern suggests that BoNT-A has different effects across goal types: passive functional goals can benefit relatively directly from focal muscle tone reduction, whereas active functional improvement typically depends on synergy between residual voluntary motor control and subsequent task-oriented training (41, 50).

This extends a methodological question: in populations with high PSS heterogeneity, using overall goal attainment rates or single functional endpoints as primary efficacy indicators without adequately considering functional goals and baseline functional status may make it difficult to distinguish between limited intervention efficacy and dilution of efficacy caused by a mismatch between goal type or baseline functional status and the intervention (45, 51). When sample sizes are insufficient to support prospective stratification, incorporating goal type as a covariate into statistical models is one alternative approach that balances feasibility with analytical completeness (31, 45).

### 5.3 Longitudinal evidence for goal-oriented treatment and research gaps

The ULIS cohort series consistently shows that incorporating personalized, explicit functional goals into the BoNT-A treatment pathway allows more precise and comprehensive assessment of clinical benefit in routine PSS management (29). For example, GAS can turn individualized goals into measurable outcomes and capture benefits that are difficult to express using traditional muscle tone scales, such as improved comfort when dressing, decreased pain during activities, and greater use of walking aids or movement for functional tasks (29, 44, 52, 53). However, existing research more strongly supports the importance of including defined goals in spasticity management than it directly supports differentiated management approaches for different goal types. When studies report generic overall GAS attainment rates, the respective contributions of different goal categories are often poorly disentangled (31). ULIS-III research indicates that patients can adjust their goals according to changes in functional status, care needs, and life roles over repeated BoNT-A treatment cycles. The relationship between functional goals and functional status indicates that functional goals may be important clinical indicators of a patient’s treatment stage rather than direct measures of their true potential or needs (29). A specialized longitudinal analysis published in 2026 further demonstrated that, even among patients with longer disease duration, some patients could continuously achieve passive functional and ease-of-care improvements across multiple treatment cycles (11), suggesting that judging the “improvement window” solely on the basis of post-stroke time may be inadequate (54). However, there is currently no clear evidence to answer several key questions. When patient goals shift from passive to active function, the corresponding adjustments to rehabilitation training and BoNT-A injection strategies remain unclear. When active functional goals remain unattained, the timing and basis for shifting toward pain control or contracture-prevention goals also lack systematic research. For patients with longer disease duration who still demonstrate passive functional benefits, maintenance treatment and follow-up-cycle arrangements similarly require further exploration. These questions exceed the explanatory scope of traditional time-based staging and single-cycle goal-setting tools, providing a basis for constructing functional goal-oriented stratification frameworks (37, 49). The clinical operability of functional goal-oriented stratification frameworks depends not only on establishing goal classification systems but also on clarifying the matching relationships between different interventions and specific functional goal types. The following sections systematically review the evidence for major interventions under different functional goals and their integration logic.

Functional goal-oriented stratification should therefore not be equated with GAS. The former is an overarching decision framework that organizes the goal domain, current clinical phenotype, attainability, and intervention matching, and defines what requires reassessment as phenotype or priorities change. GAS is embedded within this process as an individualized goal-setting and outcome-measurement tool: it converts a selected goal into explicit attainment levels but does not itself select the goal domain, treatment, or timing of goal transition. Clinicians first characterize phenotype and feasibility, select the goal domain, use GAS to define observable anchors, match the intervention, and reassess phenotype, relevance, and attainment across treatment cycles (29, 48).

## 6 Correspondence between interventions and functional goals

Post-stroke spasticity management has gradually evolved from simply reducing muscle tone toward comprehensive interventions oriented by functional goals. Clarifying the relationships between interventions and functional goals helps explain why muscle tone reduction does not necessarily translate into functional improvement and provides a therapeutic rationale for functional goal-oriented stratification frameworks.

### 6.1 Evidence for major interventions across functional goals

BoNT-A has better evidence of efficacy than most other options for treating focal spasticity. Its principal established action is suppression of acetylcholine release at extrafusal neuromuscular junctions, thereby decreasing overactivity in the target muscle. BoNT-A may also affect gamma-motor endings on intrafusal fibers, reducing muscle-spindle sensitivity and Ia/II afferent input; pilot clinical and neurophysiological evidence suggests that this may modulate spinal reflex circuits and may be accompanied by brainstem and cortical sensorimotor reorganization (55, 56). These remote effects are best interpreted as indirect consequences of altered peripheral afferentation; a direct, clinically meaningful central antispastic action has not been established (56). In patients with post-stroke upper-limb spasticity, systematic reviews and sequential meta-analyses indicate that BoNT-A improves MAS scores and passive range of motion (42). ICF-based systematic reviews also suggest that the evidence for BoNT-A is primarily focused on the body-functions-and-structures level and is relatively weak at the activity and participation levels (43). Patterns of BoNT-A use differ across countries and regions (57); however, Asian multicenter experience emphasizes the importance of individualized goal setting and multidisciplinary collaboration. Thus, BoNT- A is highly compatible with passive functional goals, including pain relief and postural improvement (41, 44, 50), and may support contracture prevention when modifiable neural muscle overactivity remains. It generally cannot reverse passive range-of-motion loss caused by fixed structural contracture; however, when residual neural muscle overactivity contributes to pain, abnormal posture, hygiene problems, or care difficulty, BoNT-A may retain goal-specific value even in a limb with established structural shortening. For example, in severe spasticity with predominantly fixed contracture, clinicians should first establish whether residual dynamic neural overactivity or usable voluntary movement remains. When structural restriction dominates, GAS anchors should prioritize less pain during care, easier hygiene or dressing, improved resting posture, or reduced caregiver assistance rather than restoration of passive range of motion or active function. BoNT-A should be considered only when residual neural overactivity is mechanistically linked to the selected outcome. It is less well suited as an adjunctive treatment for active functional goals; it creates only a limited training window and should not be used as a stand-alone functional recovery intervention (41, 58). Orthotics, serial casting, and stretching are used primarily for passive functional goals, including postural management and contracture prevention, to support sustained low-load tissue stretch, joint alignment, and soft-tissue extensibility. Systematic reviews suggest that continuous low-load stretching assisted by an orthosis may help maintain passive wrist ROM, but the evidence is limited (59, 60). For patients with active functional goals, constraint-induced movement therapy (CIMT) and task-oriented training are the primary rehabilitation approaches. A network meta-analysis including 44 randomized controlled trials and 1,779 participants demonstrated that CIMT with trunk restraint produced greater improvements in Fugl-Meyer Assessment Upper Extremity (FMA-UE) and Action Research Arm Test (ARAT) scores than control interventions (61) and may also contribute to independence in activities of daily living. Patients must retain some active motor capacity, selective motor control, and the ability to execute the task for both CIMT and task-oriented training. In patients with severe paralysis, poor sensation, significant pain, or cognitive impairment, active functional goals may not be achievable; in such cases, treatment priorities should shift toward passive functional goals, including care facilitation, pain relief, postural improvement, and contracture prevention (19, 37). In addition, functional electrical stimulation (FES), robot-assisted training, exoskeleton training, and noninvasive brain stimulation technologies are playing an increasingly important role in post-stroke motor function rehabilitation. These approaches are theoretically more appropriate for patients who have some remaining active motor potential. The optimal intervention timing and precise relationships among muscle function, neuroplastic processes, and functional goals can be established only by further research (41, 62, 63). Although the above interventions each have indications and limitations when applied individually, the complexity of PSS means that most patients require multimodal treatment. Therefore, it is necessary to examine the quality of existing multimodal treatment evidence and its integration logic under goal-oriented frameworks.

### 6.2 Goal-oriented integration of multimodal therapy

The fundamental concept of multimodal intervention integration is that muscle tone reduction relates to, but is not the same as, functional goal achievement. When the primary goals of treatment are active functional improvement, BoNT-A usually needs to be combined with task-oriented training, CIMT, gait training, FES, or robot-assisted training. However, for individuals with passive functional goals, such as pain control, postural management, or contracture prevention, BoNT-A, stretching, orthoses, serial casting, positioning, and caregiver education are more commonly used. “Functional improvement” is an outcome of PSS management rather than a single outcome; stratification according to precise functional objectives may facilitate comparative evaluation of the clinical value of different interventions (8, 41). Demetrios et al. conducted a Cochrane systematic review indicating that adjunctive BoNT-A in addition to multidisciplinary rehabilitation measures may benefit some patients (64). However, the included studies showed considerable heterogeneity in baseline patient status, treatment content, and outcome indicators; relatively few assessed functional goal type as a prospective stratification factor for goal-specific efficacy (45, 65). In the ULIS-III prospective real-world cohort study, passive functional goals were reached more often than active functional goals, whereas achieving an active functional goal additionally depended on patients’ baseline motor function (29). This finding highlights the relevance of functional goal types to patients’ motor reserve, treatment achievability, and combined intervention needs. The InTENSE randomized controlled trial showed no group differences in goal attainment or upper-limb activity when structured motor training was compared with home exercise manuals in the context of BoNT-A (66, 67). Thus, in patients with chronic post-stroke spasticity, baseline motor potential, goal type, training dose, and outcome-measure sensitivity may jointly determine whether active functional benefits can be detected after adding training to BoNT-A. Future studies should prospectively stratify patients by functional goal type and baseline characteristics, including available voluntary motor capacity and potential for task participation, to delineate subgroups that truly benefit from specific interventions (45).

Based on the above goal-oriented perspectives, this article summarizes the integration logic for PSS multimodal treatment in Table 2. In summary, distinct relationships can be established between interventions and functional goals: passive functional goals usually have a direct goal-effect relationship with focal muscle tone reduction or postural management, whereas the achievement of active functional goals is primarily determined by multimodal treatment and available baseline motor potential. The following section discusses the potential benefits of this framework based on the evidence reviewed above, including PSS clinical heterogeneity, the limitations of time-based staging, the rationale for functional goal-oriented stratification, intervention-goal correspondence, current evidence limitations, and future research directions.

**Table 2:** Integration logic for PSS multimodal treatment from a functional goal-oriented perspective.

Functional goal or dimension	Goal content	Multimodal integration logic	Relevant interventions	Notes for stratified understanding
Active function/improvement	Improving grasp. Feeding, transfers. Walking, and other active task performance	Muscle tone reduction alone is typically insufficient: its value lies in creating conditions for motor execution and subsequent task-oriented training (41, 50)	BONT-A. task-oriented training, CIMT, gait training, FES. robot-assisted therapy	Active functional gains depend more on baseline motor control, residual voluntary movement, training dose, and task specificity [38. 48]
Passive function/improvement	Improving dressing. Hygiene, positioning, and care convenience	Functional improvement is primarily reflected in limb posture, local muscle tone, and care scenarios, not necessarily active motor capacity (8, 11)	BoNT-A, stretching, orthoses, positioning, caregiver education	Passive goals are generally easier to achieve; outcomes should be distinguished from active function scales [11, 38
Pain control	Reducing spasticity-related pain, stretch pain, and discomfort from abnormal posture	Pain may arise from muscle overactivity, soft-tissue stretch, postural abnormality, or joint restriction, requires a multidimensional understanding (52)	BoNT-A, stretching. Positioning, orthoses, analgesic strategies	Distinguish spasticity-related pain from shoulder pain, joint pain, and central pain (52)
Contracture prevention	Maintaining passive range of motion and soft-issue extensibility	BONT-A targets residual neural muscle overactivity but cannot reverse fixed shortening: structural restriction requires multimodal management (54)	Stretching, orthoses, serial casting BONT-A, home exercise, follow-up	Assess spastic myopathy, residual dynamic deformity. Soft-tissue shortening, and fixed. Structural contracture (54)
Posture and care/goals	Improving abnormal limb position. Sitting/standing posture, skin pressure, hygiene, and care difficulties	These goals emphasize treatment impact on daily life and caregiver burden, reflecting an expanded concept of function in PSS management (8, 34, 73)	BONT-A positioning, orthoses, caregiver education, multidisciplinary management	Evaluation should extend beyond muscle tone scores to include caregiver burden, comfort, hygiene, and daily management feasibility (8)

This table summarizes the integration logic for multimodal PSS treatment from a functional goal-oriented perspective. The listed intervention elements represent components commonly reported in the literature. BoNT-A, botulinum toxin type A; CIMT, constraint-induced movement therapy; PSS, post-stroke spasticity.

## 7 Discussion

### 7.1 Potential advantages of functional goal-oriented stratification relative to time-based staging

Integrating the preceding evidence, functional goal-oriented stratification as a complementary dimension to time-based staging may offer several important advantages. This does not diminish the value of time- based staging; rather, it highlights the incremental contribution of functional goal-oriented stratification to individualized decision-making. Traditional time-based staging frameworks are often implicitly assumed to represent proxy indicators of patients’ overall recovery status in clinical practice. However, patients at the same time point may have vastly different core demands: some prioritize improving active functions such as grasping and walking, whereas others focus more on ease of care, pain relief, or contracture prevention (6, 8). Functional goal-oriented stratification uses patients’ currently achievable functional goals as one decision dimension (31). Patients sharing the same goal type may nevertheless differ substantially in active motor performance, neural and non-neural contributors to movement restriction, pain, mobility, and care dependency; these features must therefore be assessed alongside goal type when determining treatment feasibility and priority. This framework may also improve the alignment between interventions and functional goals. Converging evidence suggests that BoNT-A is particularly effective for passive functional goals and postural correction and may support contracture prevention when a modifiable neural component remains. Active functional improvement is observed following treatment only when combined with task-oriented training or CIMT to yield synergistic effects (58, 68). This framework is consistent with patient-centered principles of rehabilitation and aligns with the individualized goal-setting approach recommended by the ICF framework and the 2025 ESO motor rehabilitation recommendations (33, 35). The inclusion of functional goals in BoNT-A delivery is also supported by Asian multicountry clinical practice experience (57). Overall, systematic reviews of multimodal interventions have also shown that incorporating BoNT-A with task-oriented training is a step toward maximizing functional rehabilitation outcomes (34). Additionally, this framework helps avoid the misjudgment that the “improvement window” has closed. Recent longitudinal studies suggest that even patients with longer disease duration can continue to show passive functional improvements across multiple treatment cycles (54). The American Heart Association (AHA) scientific statement also emphasizes that PSS intervention should be embedded in coordinated multidisciplinary stroke care pathways rather than focusing solely on early time windows (6). Using functional goal type together with the current clinical phenotype can help assess treatment achievability without relying exclusively on time labels. Functional goal-oriented stratification does not replace time-based staging but rather introduces functional status and treatment priorities as complementary dimensions, shifting the core question of clinical decision-making from “Which time stage is the patient in?” to “Does spasticity affect function or care, what problems do patients currently most need to address, and can available interventions achieve these goals?” (1, 31). However, these potential advantages are primarily based on theoretical inference and indirect evidence from observational studies. This framework still faces numerous methodological and empirical limitations in clinical application and requires objective evaluation.

### 7.2 Limitations of current evidence and future research directions

Although the advantages described above support the rationale for functional goal-oriented stratification, no prospective randomized controlled study has directly compared clinical outcomes under this framework with those under time-based staging. Current evidence is drawn mainly from observational studies, real- world cohorts, and post-hoc analyses, so its explanatory value remains uncertain. Definitions of active and passive functional goals, GAS anchor settings, and attainment criteria vary across studies, which hampers comparability (29, 45, 69). The reliability and validity of GAS as an efficacy measure for spasticity treatment also require further study. Its use in randomized trials has been described as insufficiently standardized, with inconsistent anchor definitions and unclear endpoint criteria (70). GO-FAST-type tools have been used for PSS goal setting and efficacy assessment, but their reliability and validity in multicenter and cross-cultural studies require additional validation (37, 47, 69). A core outcome set development study for adult spasticity was initiated in 2025 to provide consensus-based guidance on standardized assessment systems (71). Broad adoption of appropriate consensus instruments could improve consistency in goal classification across multicenter studies. Regarding dynamic goal evolution, ULIS-III and other longitudinal studies suggest that goal types can change with functional status (29, 49). However, the drivers, predictive indicators, and optimal timing of such transitions remain unclear, limiting the framework’s use in multi-cycle clinical management (31, 72). It is unknown whether passive-to-active transitions have predictable clinical indicators or whether the timing and method of adjusting an unattained active goal affect subsequent outcomes; these questions lack systematic longitudinal research (31). Although the 2026 ULIS-III multi-cycle passive-function analysis provided preliminary evidence of sustained benefits across cycles, its observational design cannot answer these causal questions; the authors identified the absence of a control group and randomized design as major limitations (11). Evidence on intervention matching also derives mainly from indirect analyses and subgroup observations. One systematic review found substantial misalignment between randomized trials of BoNT-A and spasticity-management guidelines: individualized goal setting was reported in only 25% of studies, multidisciplinary management in 11.5%, and follow-up longer than 6 months in 7.7% (45). This misalignment may help explain why existing trials have struggled to detect functional benefits. Negative results from BEST and InTENSE cannot readily distinguish limited intervention effects from insufficient baseline functional potential for goal attainment (40, 66, 67). Future trials should prospectively stratify participants by goal type and baseline characteristics, such as residual voluntary motor capacity and potential for task participation, to identify intervention combinations that benefit specific subgroups (6, 19, 37). PSS research also insufficiently integrates patient and caregiver perspectives. A multidisciplinary Delphi consensus statement identified gaps in patient and caregiver knowledge and education, as well as clinicians’ limited understanding of individualized needs, as core barriers to high-quality spasticity management (73). Patient-experience research suggests that the effects of spasticity on daily life are more complex than tone scores indicate; treatment goals should therefore incorporate daily living needs and care priorities (72, 74). This perspective remains underrepresented in PSS research and framework design. Integrating time-based staging with functional goal-oriented stratification can provide a clearer analytical framework for these questions. The two approaches are complementary:

time-based staging describes disease-course context, whereas functional goals describe current needs. This review therefore proposes a conceptual framework combining both dimensions for PSS management, as shown in Table 3.

**Table 3:** Conceptual framework integrating time-based staging and functional goal-oriented stratification for PSS management.

Functional goal or time phase	Early (0–3 months)	Subacute (3–6 months)	Early chronic (6–12 months)	Chronic (>12 months)	Key assessment points
Active functional goals	Identify residual voluntary movement, selective motor control, and task participation potential (25)	Determine achievability of active-function recovery and assess task-specific training needs (33, 61)	Distinguish insufficient training from insufficient motor reserve and evaluate whether goals remain attainable (21, 66, 67)	Focus on maintenance of task capacity, compensatory strategies, and long-term participation goals (29, 54)	Does the patient retain usable voluntary movement? Are active goals clinically attainable at the current stage?
Passive functional goals	Early identification of risks related to positioning. Hygiene, dressing, and care difficulties (17, 30)	Monitor ease of care, limb posture, and changes in passive range of motion (28, 40, 59)	Evaluate whether care goals persist and whether adjustment is needed (11, 29)	Focus on long-term caregiver burden, hygiene, skin protection, and comfort (8, 11)	Have care difficulties or postural abnormalities emerged or worsened? Should intervention intensity be adjusted?
Pain-control goals	Identify pain sources and distinguish spasticity-related pain from other pain types (13, 52)	Analyze relationships among pain, tone, postural abnormality, and soft-tissue stretch (34, 52)	Assess the risk of pain chronification and multifactorial mechanisms (52, 75)	Focus on chronic Has the pain pain management, comfort, and quality of life (52, 73)	Hasthepain sourcebeen differentiatedas spastic, structural,or central? Has painseverity affectedfunction orcare feasibility?
Contracture-prevention goals	Early screening for reduced joint range of motion and abnormal postural trends (17)	Assess residual neural muscle overactivity or dynamic deformity and the reversibility of passive restriction (54, 59)	Evaluate spastic myopathy, structural progression, and joint alignment (54, 59)	Manage fixed. Structural contracture, posture, and care feasibility (34, 54)	Is a residual dynamic component present? Is passive restriction reversible? Do goals match residual movement and structural status?

This conceptual framework combines time-based staging with functional goal-oriented stratification. Time phase provides context but does not determine goal priority; goals should be reassessed during each treatment cycle according to phenotype, achievability, and patient or caregiver needs.

To illustrate the integration of time-based staging and functional goal-oriented stratification, Figure 2 displays the dynamic application of this framework in clinical practice. The horizontal axis provides the disease-course context through time-based staging, whereas the vertical axis reflects patients’ current treatment needs through functional goal types. Different border thicknesses identify high-priority goals at each time stage, and arrows indicate that functional goals can evolve with patients’ functional status. This framework may help clinicians balance disease-course stage and functional status in multi-cycle treatment decisions.

**Figure 2:**
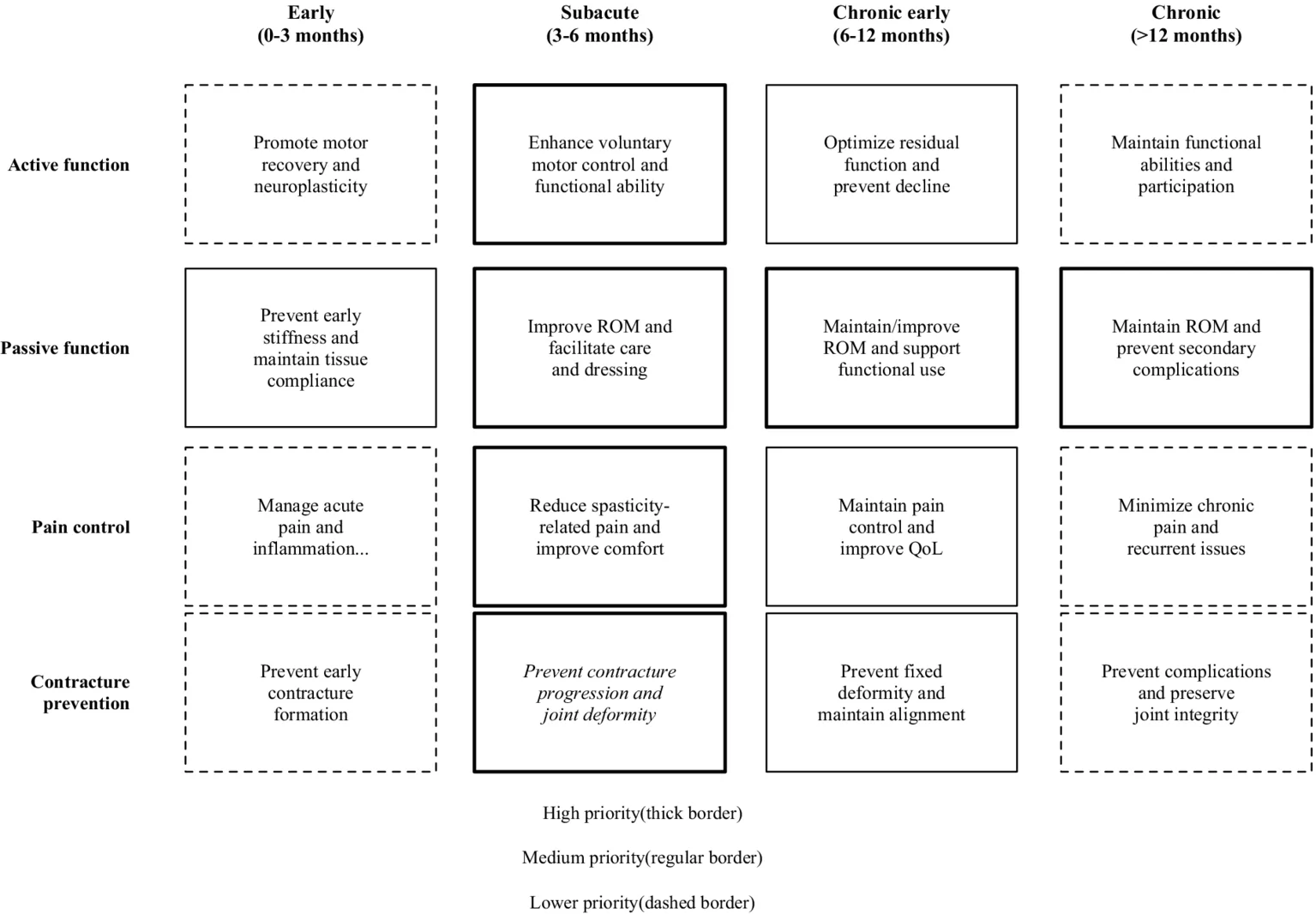
Integration framework of time-based staging and functional goal-oriented stratification in PSS management. This framework integrates time phases and functional goal categories. Time phases provide disease-course context rather than fixed treatment priorities. High-, medium-, and low-priority designations, including bold borders for high-priority goals, are illustrative rather than prescriptive. Priorities should be reassessed during each treatment cycle according to current clinical phenotype, goal achievability, and patient or caregiver needs; arrows indicate possible dynamic goal evolution as functional status changes. BoNT-A, botulinum toxin type A.

## 8 Conclusion

PSS management should move from a purely time-centered framework toward individualized decision- making that integrates time-based staging with functional goal-oriented stratification. Time-based staging remains valuable for research standardization, prognostic framing, and clinical pathway development, but it does not sufficiently explain why patients at similar post-stroke intervals may have different functional status, treatment priorities, and intervention responses. This review proposes each patient’s currently achievable functional goal type as a complementary decision dimension rather than a complete clinical phenotype. Available evidence indicates systematic differences between active and passive goals in patient distribution, baseline functional status, intervention response, and outcome evaluation (28, 31, 40). Functional goals are not static labels; they may evolve with treatment progression, neuromotor recovery potential, and changing care needs (11, 29, 49).

Future research should prioritize three areas. First, functional goal classification and assessment tools should be standardized, preferably through multidimensional International Classification of Functioning, Disability and Health-based systems that can be applied across studies, cultures, and multicenter settings (45, 69, 70). Second, prospective randomized trials should stratify patients by functional goal type, especially in studies of robot-assisted training, neuromodulation, and multimodal rehabilitation, to identify subgroups most likely to benefit from specific intervention combinations (45, 50, 65). Third, longitudinal studies should define predictive indicators of goal evolution, clarify when treatment goals should shift across cycles, and establish decision rules for multi-cycle management (11, 49).
